# Insights into the Pathogenesis and Development of Recombinant Japanese Encephalitis Virus Genotype 3 as a Vaccine

**DOI:** 10.3390/vaccines12060597

**Published:** 2024-05-30

**Authors:** Jae-Yeon Park, Hye-Mi Lee, Sung-Hoon Jun, Wataru Kamitani, Onnuri Kim, Hyun-Jin Shin

**Affiliations:** 1 College of Veterinary Medicine, Chungnam National University, Daejeon 34134, Republic of Korea; wodus5818@cnu.ac.kr (J.-Y.P.); hyemi0728@cnu.ac.kr (H.-M.L.); onr3348@cnu.ac.kr (O.K.); 2 Electron Microscopy & Spectroscopy Team, Korea Basic Science Institute, Cheongju 28119, Republic of Korea; jsh100@kbsi.re.kr; 3 Department of Infectious Diseases and Host Defense, Gunma University Graduate School of Medicine, Maebashi 371-0034, Japan; wakamita@gunma-u.ac.jp; 4 Research Institute of Veterinary Medicine, Chungnam National University, Daejeon 34134, Republic of Korea

**Keywords:** Japanese encephalitis virus 3, Nakayama, infectious clone, cDNA

## Abstract

Japanese encephalitis virus (JEV), a flavivirus transmitted by mosquitoes, has caused epidemics and severe neurological diseases in Asian countries. In this study, we developed a cDNA infectious clone, pBAC JYJEV3, of the JEV genotype 3 strain (EF571853.1) using a bacterial artificial chromosome (BAC) vector. The constructed infectious clone was transfected into Vero cells, where it exhibited infectivity and induced cytopathic effects akin to those of the parent virus. Confocal microscopy confirmed the expression of the JEV envelope protein. Comparative analysis of growth kinetics revealed similar replication dynamics between the parental and recombinant viruses, with peak titers observed 72 h post-infection (hpi). Furthermore, plaque assays demonstrated comparable plaque sizes and morphologies between the viruses. Cryo-electron microscopy confirmed the production of recombinant virus particles with a morphology identical to that of the parent virus. Immunization studies in mice using inactivated parental and recombinant viruses revealed robust IgG responses, with neutralizing antibody production increasing over time. These results showcase the successful generation and characterization of a recombinant JEV3 virus and provide a platform for further investigations into JEV pathogenesis and vaccine development.

## 1. Introduction

Japanese encephalitis (JE) is an inflammatory brain disease caused by the Japanese encephalitis virus (JEV) [1]. JEV is a zoonotic mosquito-borne virus, commonly associated with viral encephalitis [2]. It is the leading cause of viral encephalitis in Asia, the western Pacific, and northern Australia [3,4]. In endemic regions, JE primarily affects children under the age of 15 but travel-associated cases can occur at any age in the absence of protective immunity to JEV [5]. The virus persists within an enzootic cycle, primarily involving Culex mosquitoes and various amplifying vertebrate hosts, such as aquatic birds and domestic pigs [6]. Humans are considered terminal hosts, as they typically do not reach sufficiently high levels of JEV in their blood circulation to transmit the virus to mosquitoes during feeding [7].

JEV belongs to the *Flaviviridae* family, *Flavivirus* genus, and is transmitted by mosquitoes [8]. Phylogenetic analysis of E genes classified JEV into five distinct genotypes (I, II, III, IV, and V) and genotype III was the most widespread genotype in endemic countries [6]. Nowadays, genotype I has become the dominant strain and genotype V, considered the ancestor of other genotypes, has re-emerged in South Korea [9]. The JEV is an enveloped virus with a single-stranded positive-sense RNA genome approximately 11kb in length [10]. Its genome comprises a single open reading frame (ORF) flanked by 5′ and 3′ untranslated regions (UTR) [11]. This RNA genome possesses a type I cap structure at its 5′ terminus but lacks a polyadenylation tail [12]. Within the ORF, a large polyprotein is encoded, consisting of three structural proteins and seven non-structural proteins, arranged in the genome as follows: C-prM-E-NS1-NS2A-NS2B-NS3-NS4A-NS4B-NS5 [13]. After translation, the polyprotein undergoes cleavage by NS2B-NS3 viral proteases, a process essential for viral replication [14].

The evolution of reverse genetics methodologies has significantly impacted the investigation of positive-sense RNA viruses [15]. Notably, viral genes have been shown to exhibit toxicity to *E. coli* during the cloning process [16]. Furthermore, full-length cDNA clones may display instability during propagation in bacteria, leading to cloning failures or mutations due to viral genome instability [17]. Various studies have proposed methodologies to alleviate the toxicity of full-length constructs in bacteria, including the use of low-copy number plasmids or bacterial artificial chromosomes (BACs) [18]. Additionally, the introduction of bacterial intron sequences into the viral genome has been shown to suppress the expression of toxic regions [19].

In addition, in the conventional RNA-initiated approach, cells undergo transfection with RNA transcripts derived from infectious cDNA clones [20]. Alternatively, synthetic viruses can be engineered through direct transfection of RNA transcriptomes. These methodologies encompass the in vitro transcription of an infectious RNA genome from a full-length cDNA template containing the viral genome under the governance of a prokaryotic RNA polymerase promoter, such as T7 or SP6 [21]. Another avenue involves the direct transfection of DNA plasmids to generate an infectious genome. This methodology encompasses the development of a comprehensive full-length infectious cDNA clone, housing the entirety of the viral genome [22]. The clone is meticulously constructed with a eukaryotic polymerase promoter, notably the cytomegalovirus (CMV) promoter, situated at its 5′ terminus, in conjunction with the hepatitis delta virus ribozyme (HDVr) sequence. At the 3′ terminus of the viral genome, a polymerase II terminator serves as the concluding element [17].

In instances where issues arise from the utilization of bacteria, alternative approaches, such as bacterium-free methods, can be employed. The utilization of the infectious subgenomic amplicons (ISA) approach plays a pivotal role in facilitating the generation of recombinant viruses. This method entails the precise transfection of overlapping PCR fragments, which collectively span the entirety of an RNA virus genome. These PCR fragments are distinctly characterized by the presence of a CMV promoter at their 5′ terminus and the HDVr sequence at their 3′ terminus. Following this delineation, a polymerase II terminator and polyadenylation signals are incorporated [23]. Another approach involves the utilization of circular polymerase extension cloning (CPEC) in conjunction with a high-fidelity DNA polymerase. This technique entails the joining of several RT-PCR amplicons that have overlapping ends with a vector fragment, resulting in the formation of a circular end product [24].

In this study, we developed a new full-length cDNA infectious clone for JEV genotype 3 using cDNA-based technology. We demonstrated that our full-length cDNA infectious clone produces progeny virus particles when transfected into susceptible Vero cells. The recovered viruses exhibited similar characteristics to the parent virus, including growth kinetics, plaque morphology, protein expression, and antigenicity. Furthermore, we utilized a standard mutagenesis procedure to introduce a point mutation into the BAC construct, serving as a genetic marker. This allowed us to confirm the generation of recombination viruses. Our findings hold significant potential for conducting various studies on JEV genotype 3, including basic research, diagnostics, and the development of new vaccines.

## 2. Materials and Methods

### 2.1. Cell Culture, Virus Preparation, and Antibodies

African green monkey kidney cell lines (Vero; CCL-81, ATCC, Manassas, VA, USA) were maintained in Minimum Essential Media (MEM, Gibco, Waltham, MA, USA) supplemented with 10 mM HEPES (Gibco), 1× Antibiotic-Antimycotic (Gibco), and 10% fetal bovine serum (FBS; Gibco) at 37 °C with 5% CO_2_. The genotype 3 of JEV (JEV3; GenBank number EF571853.1) was provided by Dr. Wataru Kamitani and infectious clones were propagated in Vero cells. The following antibodies were a mouse monoclonal antibody (mAb) 4G2 and a goat anti-mouse antibody conjugated with Alexa Fluor 488, both purchased from Thermo Fisher Scientific (Waltham, MA, USA).

### 2.2. cDNA Synthesis and Cloning

**Figure 1:**
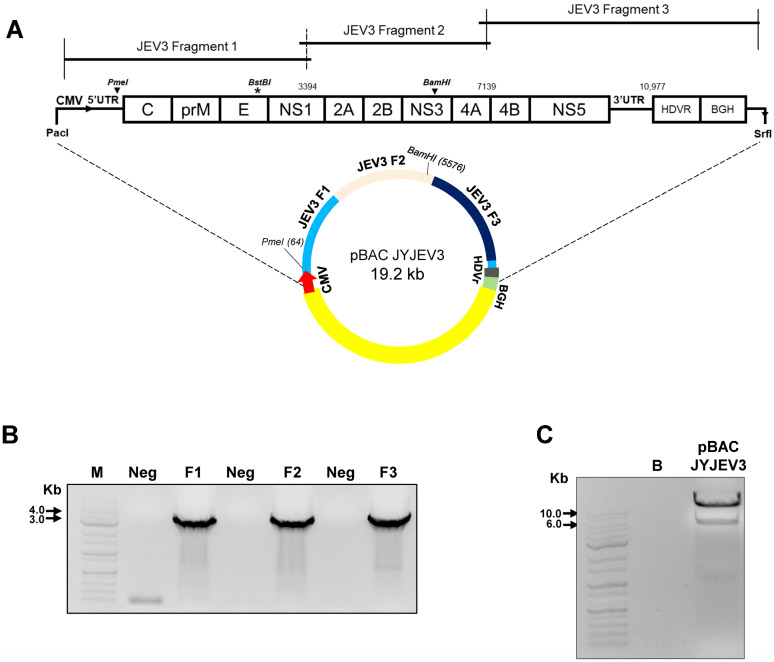
Schematic diagram of the construction of an infectious cDNA clone of JEV3. (**A**) Strategy for constructing the full-length cDNA clone of JEV3. Three cDNA fragments (F1 to F3) were synthesized from the plasmid template (Supplementary Table S1). (**B**) The individual fragments were amplified and confirmed on an agarose gel. Three fragments were assembled to form the full-length cDNA clone of JEV3 (pBAC JYJEV3). (**C**) Lane 1 contains the DNA size marker. Lane 2 is a blank control. Lane 3 contains pBAC JYJEV3 DNA digested with the restriction enzymes PmeI and BamHI. The enzymatic restriction analysis results indicated that the full JEV3 viral genome exists in the BAC vector. M, marker; B, blank; F1, JEV3 Fragment 1; F2, JEV3 Fragment 2; F3, JEV3 Fragment 3. Black triangles indicated single-cut enzyme sites (PmeI and BamHI); Asterisk indicated silent mutation.

Viral RNA was extracted from viral stocks using the Ribospin vRD kit (Geneall, Seoul, Republic of Korea) and cDNA synthesis was performed using the TOPscript™ RT DryMIX dT18/dN6 plus (Enzynomics, Daejeon, Republic of Korea). The JEV full genome was separated into 8 fragments and PCR amplification was carried out using virus-specific primer (Table 1). Each fragment was cloned into the pGEMT easy vector (Promega, Madison, WI, USA). The fragment was then fused using the In-Fusion^®^ Snap Assembly Master Mix kit (Takara, Tokyo, Japan) to create three larger fragments: a JEV fragment 1 (A + B + C), JEV fragment 2 (D + E + F), and JEV fragment 3 (G + H) (Figure S1). They were cloned into pBAC vectors using Infusion protocols. The final plasmid containing the full-length cDNA of JEV with a CMV promoter and a hepatitis delta virus ribozyme (HDVr) was added at the 5′ and 3′ end (Figure 1).

### 2.3. Transfection and Propagation of Recombinant JEV3

Plasmid pBAC JYJEV3, containing the full-length cDNA of JEV, was amplified in *E. coli* HST08 premium (Takara, Japan) and purified using NucleoBond Xtra Maxi (Macherey-Nagel, Düren, Germany) following the manufacturer’s instructions. Vero cells were seeded in a 6-well plate and the transfection mixture was prepared with 200 µL of Opti-MEM medium (Gibco, USA) containing TranslT-LT1 (Mirus, Madison, WI, USA) and 6 μg of pBAC JYJEV3 plasmid vector according to the manufacturer’s instructions.

Cell supernatant media were then serially passaged several times in Vero cells. Briefly, once the CPE was observed as a result of the transfected or infected virus, the cell supernatants were collected and centrifuged at 5000× *g* for 10 min. The cell pellets were then freeze-thawed twice. Cell-free supernatants were diluted in a new culture medium and inoculated into Vero cells for the next passages.

### 2.4. Analysis of Confocal Fluorescence Microscopy

Vero cells were seeded in 12-well plates and subsequently infected with the rescued supernatant obtained from the pBAC JYJEV3 plasmid vector or the parental virus. After 96 h post-infection, the cells were fixed using 4% paraformaldehyde for 15 min. Subsequent to fixation, the cells underwent three washes with phosphate-buffered saline (PBS), followed by permeabilization using 0.25% Triton X-100 for 10 min and blocking in PBS containing 2% BSA for 1 h at room temperature (RT). After another round of PBS washes, the coverslips were incubated with anti-flavivirus E 4G2 primary antibodies (Millipore, Burlington, MA, USA) in PBS containing 2% BSA overnight at 4 °C. Following additional PBS washes, the coverslips were exposed to Alexa Fluor 488 (Invitrogen, Carlsbad, CA, USA) for 2 h at RT. Subsequent to further PBS washes, the nuclei were stained using Hoechst 33258 (Thermo Fisher). After a 15-min staining period, the coverslips were rinsed with PBS and mounted onto microscope slides (Optinity OptiView software 4.11.2023, Seoul, Republic of Korea).

### 2.5. Sequence Analysis of the Recombinant Virus

To determine whether the virus was recombinant or not, viral RNA was extracted from both the parent strain and recombinant strain at various passages. Based on the sequence of the JEV strain (GenBank: EF571853.1), pairs of primers were designed to amplify the E genes (Table 1). The PCR amplicon was digested with *BstBI* (NEB, Ipswich, CA, USA). Additionally, the PCR products were cloned into the pGEM T easy vector and sequenced using Sanger sequencing.

### 2.6. Cryo-Electron Microscopy (Cryo-EM) Analysis

The parental virus or the recombinant virus sample of 3 µL was deposited onto freshly glow-discharged holey carbon grids (Quantifoil R1.2/1.3 Au300, Quantifoil Micro Tools GmbH, Jena, Germnay). The grids were then rapidly frozen by plunging into liquid ethane using a Vitrobot Mark IV (Thermo Fisher Scientific Inc.) at 100% humidity and 4 °C. Image acquisition was conducted at the Korea Basic Science Institute using a Talos Arctica G2 transmission electron microscope (Thermo Fisher Scientific Inc.), which was operated at 200 kV under parallel illumination. Data were collected in the EFTEM mode with a 20 eV slit width, using a BioQuantum energy filter and a K3 direct electron detector (Gatan Inc., Pleasanton, CA, USA). The nominal magnification was set to 49,000×, resulting in a calibrated pixel size of 1.7 Å. A spot size of 3 and a 50 µm C2 aperture were used. EPU 3.6 software (Thermo Fisher Scientific Inc.) was utilized to manage data collection. Images were recorded in electron counting mode, within a defocus range of −3.5 µm for 5.0 s, at a dose rate of 18.6 e^−^/pixel/s, yielding a total dose of 50 e^−^/Å^2^.

### 2.7. Viral Titration

Vero cells were seeded in 96-well plates. Both the parental and recombinant viruses were subjected to 10-fold serial dilutions in media. These diluted virus samples were then inoculated onto Vero cells and incubated at 37 °C for 1 h. Subsequent to the removal of the inoculum, the cells were cultured in growth media at 37 °C for 5 days. Additionally, Vero cells were seeded into 6-well plates one day prior to conducting the plaque assay. Following the removal of the culture media and PBS washing, virus samples were diluted 10-fold in MEM media and 200 μL of the dilution was added to the Vero cells. The overlay medium, consisting of a mixture of 1.6% agar (BD Difco, Franklin Lakes, NJ, USA), 2× MEM (Gibco) supplemented with 1× penicillin-streptomycin (Gibco), and 10% FBS, was then prepared and 3 mL of the overlay medium was added to each well. The plates were subsequently incubated at 37 °C for 5 days. Following the incubation period, the cells were fixed with 10% formalin and stained with 0.2% crystal violet dissolved in 10% absolute methanol.

### 2.8. Mouse Immunization

The inactivation of the virus was conducted using a UV lamp and placed under the lamp at a distance of 30 cm [25]. The virus was irradiated three times for 10 min each, with equal time intervals between each irradiation. The numbers of animals used in each group are indicated in Figure 6A. Blood samples were collected from all experimental groups at intervals of 0, 21, 28, 35, 42, and 49 days post-vaccination (dpv) intervals. The Institutional Animal Care and Use Committee at the Chungnam National University approved all animal experiments under protocol number 202209A-CNU-177.

### 2.9. Enzyme-Linked Immunosorbent Assay (ELISA) Analysis

Antibody titers of JEV3-specific IgG in serum from immunized mice were determined by ELISA. Briefly, 96-well plates were coated with parent virus overnight at 4 °C and blocked with 3% skim milk for 30 min at RT. Diluted sera were added and incubated at RT for 2 h. After incubation, the inoculum was discarded and wells were washed three times with PBSt (PBS with 0.1% Triton X-100). Antigen-specific antibodies were identified using peroxidase-labeled goat-anti-mouse IgG antibody (Cusabio, Boston, MA, USA). The enzymatic activity was detected by adding 3,3′,5,5′-tetramethlylbenzidine substrate (Labis Koma, Seoul, Republic of Korea), stopping by 2 N H_2_SO_4_ (Labis Koma), and then measuring the absorbance at 450 nm a microplate reader.

### 2.10. Serum Neutralization (SN) Test

Sera collected from each experimental group of immunized mice were heat-inactivated at 56 °C for 30 min and subsequently subjected to 2-fold serial dilutions. JEV, at a concentration of 200 TCID_50_/0.05 mL, was then mixed with an equal volume of the diluted serum and incubated for 1 h at 37 °C. Vero cells were infected with 0.1 mL of each virus-serum mixture. After 1 h incubation at 37 °C, the cells were washed twice with PBS and maintained in MEM at 37 °C for 5 days. The samples were added in duplicate onto 96-well plates. SN titers were determined as the reciprocal of the highest serum dilution that resulted in the inhibition of cytopathic effect.

### 2.11. Statistical Analysis

Data are presented as mean ± standard deviation (SD). Statistical significance was calculated using the SPSS 26.0 program. A *p*-value of < 0.05 was considered statistically significant.

## 3. Results

### 3.1. Construction of the Full-Length cDNA Clone for JEV3

Viral RNA extracted from Vero cells was used to generate RT-PCR fragments (A-H) spanning the complete viral genome of JEV3, which were then individually cloned and assembled into full-length cDNA (Figure S1). Fragments were cloned and inserted into a low-copy number plasmid, pBAC, utilizing the infusion protocol. Specifically, JEV3 fragment 1 (A + B + C), JEV3 fragment 2 (D + E + F), and JEV3 fragment 3 (G + H) were inserted into pBAC vectors (Figure 1A). Subsequently, all fragments were incorporated into the pBAC vector containing the cytomegalovirus (CMV) promoter, hepatitis deltavirus ribozyme (HDVr), and bovine growth hormone (BGH) polyadenylation signal at the 5′ and 3′ ends (Figure 1B). The plasmid DNA mapped with *PmeI* and *BamHI* restriction enzymes (Figure 1C). These results demonstrate the successful generation of a complete cDNA clone of JEV3 by assembling fragments spanning its genome into a plasmid vector containing essential regulatory elements.

### 3.2. Transfection of the JEV3 Infectious Clone Plasmid Produced Infectious JEV3 Particles

**Figure 2:**
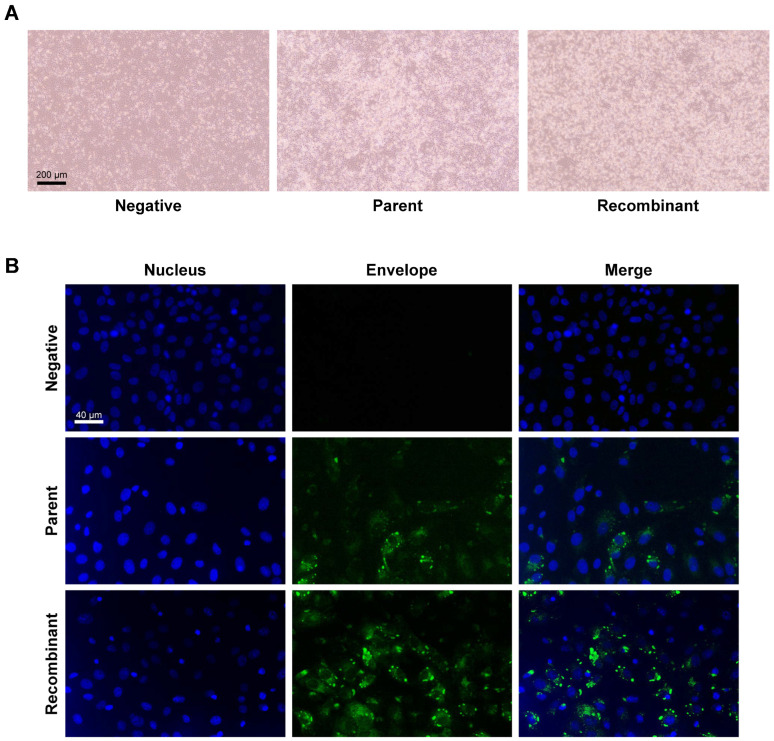
Virus confirmation: cytopathic effects and viral protein expression in Vero cells. (**A**) Vero cells were infected with the parental and recombinant viruses at an MOI of 0.01 and incubated for 3 days. Morphological changes were examined daily. Cytopathic effects with cell clumping were confirmed at 72 hpi. Scale bar, 200 μm. (**B**) For IFA, virus-infected cells were fixed at 96 hpi. IFA was performed using a mouse anti-Flavi envelope monoclonal antibody as the primary antibody. Goat-anti-mouse IgG labeled with fluorescein 488 was used as the secondary antibody (green). The nuclei were stained with Hoechst 333258 (blue). Scale bar, 40 μm.

We transfected pBAC JYJEV3 into Vero cells to evaluate the infectivity of the cDNA clone. The cytopathic effects (CPEs) of the transfected cells were monitored daily and compared with those of cells infected with the parent virus. CPE was observed in cells transfected with the infectious clone starting 4 days post-transfection, while cells infected with the parent virus exhibited CPEs at 4 days post-infection (Figure 2A). Additionally, the transfected cells were examined for viral protein expression. Confocal microscopy analysis using anti-flavivirus envelope protein antibodies revealed expression of the JEV E protein in both parent and recombinant virus-infected Vero cells (Figure 2B). In these results, transfection of the JEV3 infectious clone plasmid into Vero cells produced infectious JEV3 particles with observable cytopathic effects and viral protein expression.

### 3.3. Genetic Marker Development and Mutation Analysis of Recombinant JEV3

**Figure 3:**
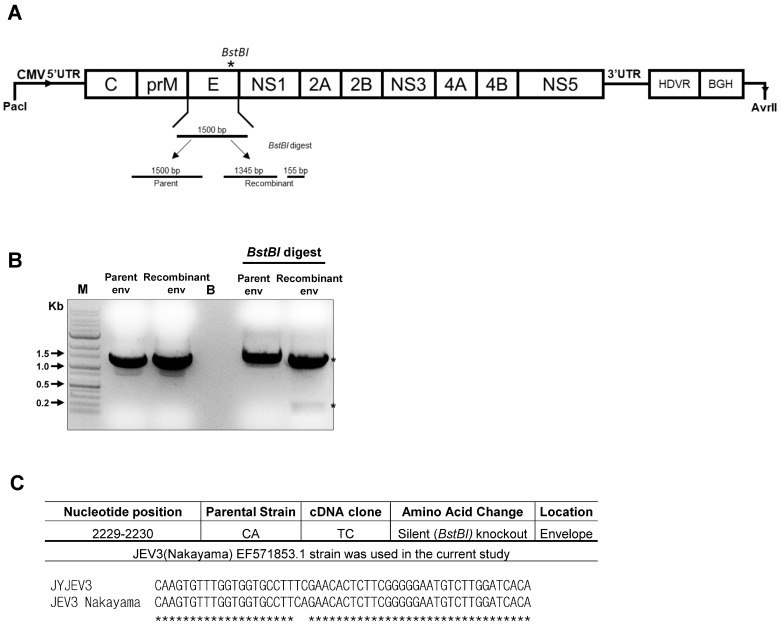
Analysis of the genetic markers of the recombinant and parental viruses. (**A**) Graphical illustration of the process of the mutant virus generation. The BstBI cleavage site was inserted in the middle of the viral E gene. (**B**) Agarose gel analysis of the mutant virus. The PCR products were digested using BstBI and loaded on an agarose gel. The expected digestion pattern described in (**A**) was confirmed. (**C**) The parental and recombinant viruses were confirmed by Sanger DNA sequencing. The amplified E gene was used for the differentiation of both virus sequences. M, marker; B, blank; env, envelope. Asterisk indicate the digested bands.

Whole-genome sequencing was used to verify the introduction of a silent mutation in the E gene using a primer-mediated point mutation to distinguish JEV3 from the parent virus. Specifically, we confirmed the generation of a silent variant with a *BstBI* site mutation from TCAGAA to TTCGAA (Figure 3A). One thousand five hundred bp fragments spanning nucleotides 978 to 2477 of the viral genome were amplified using RT-PCR from RNAs extracted from both the parental and recombinant viruses (Table 1). The RT-PCR product from the recombinant virus was cleaved into two fragments, 1345 bp and 155 bp, by *BstBI*, while that from the parent virus remained resistant to *BstBI* digestion and showed no change (Figure 3B). During the twenty-five generations, silent mutations at positions 2229 nt and 2230 nt were identified with no amino acid changes in either the parental or recombinant virus. (Figure 3C). This suggests that specific silent mutations were successfully introduced and maintained in the recombinant virus over multiple generations without resulting in any changes to the amino acid sequence.

### 3.4. Comparison of the Growth Kinetics of the Parental and Recombinant Virus in Cell Culture

**Figure 4:**
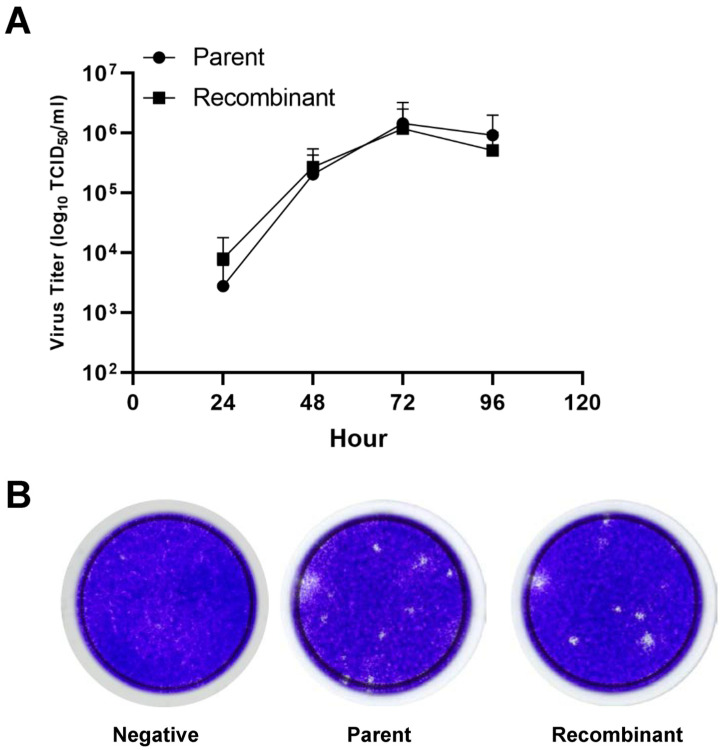
Viral growth kinetics and plaque assay. (**A**) Growth kinetics of Vero cells infected with the parental and recombinant viruses at a MOI of 0.01. Viruses were harvested at the indicated hpi and the titer was determined by TCID50 assay. Growth curves represent average titers from three independent experiments. (**B**) Plaque assays of parental and recombinant viruses. Vero cells were infected with both viruses, overlaid with agar, and stained with crystal violet at 5 dpi. The viral titers of harvested cells were determined at each time point. Data are the mean ± SD.

As we found that there was no significant difference in envelope protein expression, we compared the growth kinetics of both the parental and recombinant viruses. As shown in Figure 4A, both the parent and recombinant virus titers steadily increased between 24 and 72 hpi. The parent JEV exhibited slightly slower growth kinetics than the recombinant virus during the initial 48 hpi but eventually reached the same peak titer of 2.7 × 10^6^ TCID_50_/mL at 72 hpi. This result suggested that the replication kinetics of the recombinant virus were more or less the same as those of the parent virus (Figure 4A). Plaques were observed at 3–5 dpi and their shape and size were also the same for both viruses. (Figure 4B). This finding indicates that, despite initial differences in growth kinetics, the recombinant virus ultimately reached a similar peak titer as the parent virus, suggesting comparable replication kinetics between the two viruses.

### 3.5. JEV3 Parent and Recombinant Virus Morphology Was Revealed by Cryo-EM Analysis

**Figure 5:**
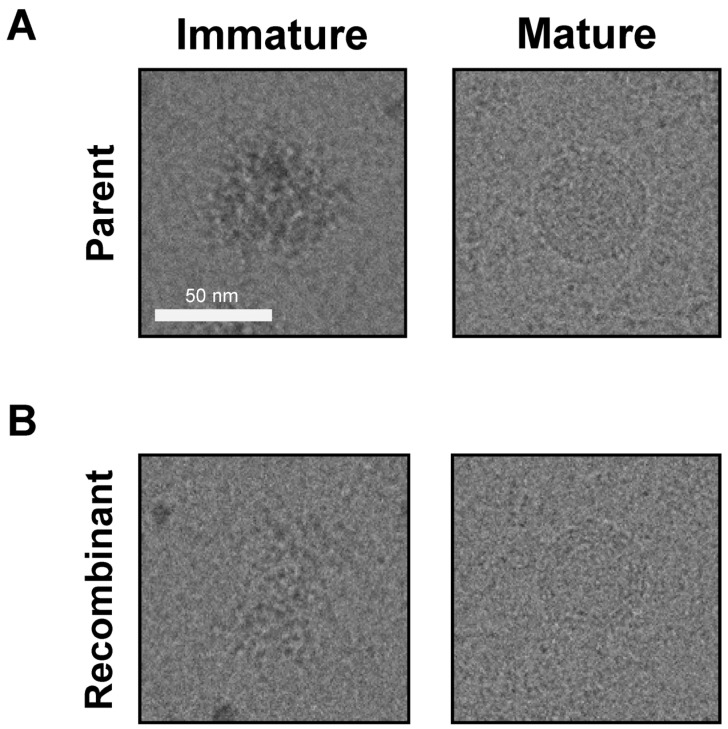
Cryo-EM images. Cryo-EM images of parental (**A**) and recombinant viruses (**B**), showing both immature (**left**) and mature virions (**right**). Scale bar, 50 nm.

Next, we investigated the morphology using cryo-EM. As shown in Figure 5, both types of immature virus particles exhibited bumpy surfaces and larger diameters than the mature particles, while mature virus particles displayed smooth spherical surfaces (Figure 5A,B). The diameter of the parental and recombinant viruses was approximately 50 nm. Cryo-EM analysis clearly confirmed that the recombinant virus was successfully produced from pBAC JYJEV3 and exhibited a morphology identical to that of the parent virus. These results suggest that cryo-EM analysis confirmed that the recombinant virus closely resembled the parent virus in morphology, exhibiting similar characteristics of immature and mature virus particles.

### 3.6. Investigation of the Immune Response in Mice

**Figure 6:**
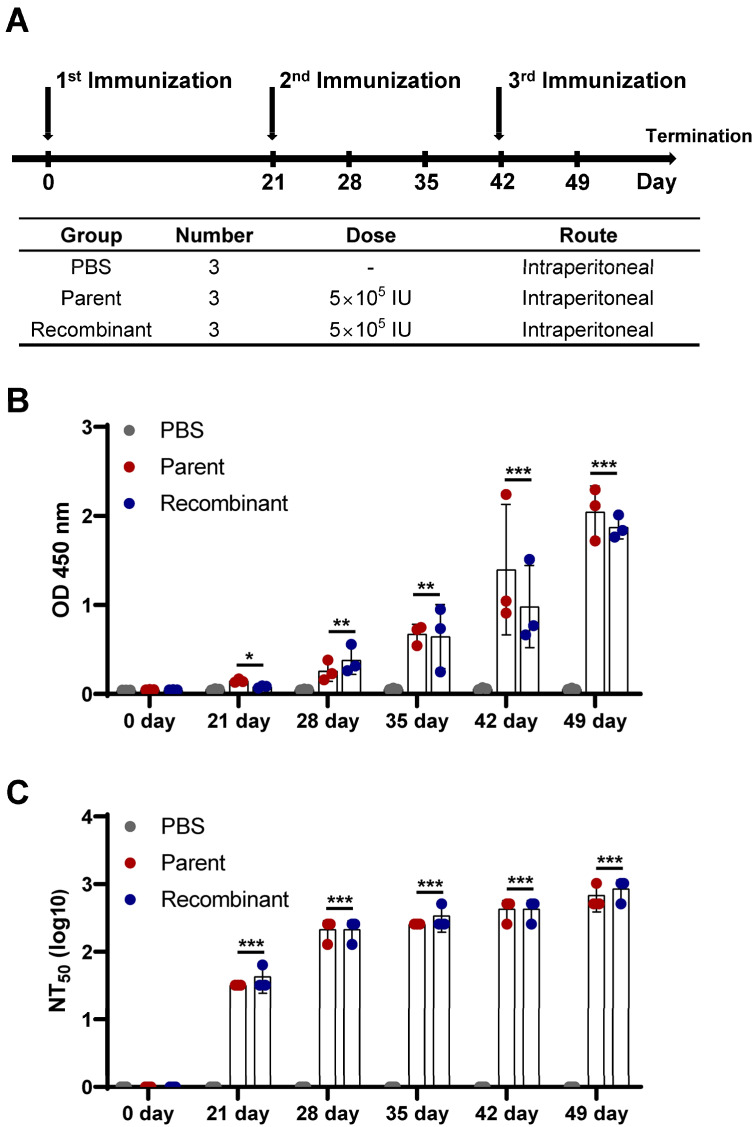
Immune responses in mice. (**A**) Schematic representation of the immunization schedule. Five-week-old mice (n = 3 per group) were immunized with PBS (negative control), parental virus, or recombinant virus. Sera were collected at 0, 21, 28, 35, 42, and 49 days after immunization. (**B**) Measurement of JEV-specific IgG titers by ELISA. (**C**) Detection of neutralizing antibody titers (NT50) in sera. The results are presented as the mean NT50 ± SD (n = 3). The groups immunized with parental and recombinant viruses were compared with the PBS group at each time point. Statistical significance is indicated as * *p* < 0.05, ** *p* < 0.01, and *** *p* < 0.001.

Five-week-old C57/BL6 mice were immunized with either the inactivated parental virus or the recombinant virus. Three mice per group were assigned to receive the parental virus, recombinant virus, or PBS as a negative control, with the experimental regimen detailed for each group (Figure 6A). IgG titers were detected in the sera from immunized mice on day 21. After the second immunization, the IgG titer increased in both the parental and recombinant virus-immunized groups (Figure 6B). The neutralizing activities were evaluated against the parent virus, as depicted in Figure 6A. The negative control group showed no neutralizing activity. Neutralizing activity was similar between both groups and continued to increase over time. The initial detection of neutralizing antibodies was observed on day 21, with titers reaching 1:32 and 1:42 for the parental and recombinant virus groups, respectively. After the second immunization, the neutralizing antibody titer increased approximately 8-fold compared to that after the first immunization; specifically, the neutralizing antibody titer reached 1:213 for the parental virus and 1:256 for the recombinant virus. Before the third immunization (days 35 and 42), a slight increase was observed in both virus groups. Finally, after the third immunization, the neutralizing antibody titers significantly increased to 1:682 and 1:853 for the parental and recombinant viruses, respectively (Figure 6C). In conclusion, immunization with both the parental and recombinant viruses led to a time-dependent increase in IgG titers and neutralizing antibody production in mice.

## 4. Discussion

The reverse genetic system is one of the most powerful tools in molecular virology as it allows for the genetic manipulation of infectious recombinant viruses from complementary DNA (cDNA) clones of the viral genome [26]. These systems have significantly contributed to virology research, vaccine development, and therapeutics [27]. However, assembling full-length cDNA clones has remained challenging due to the presence of bacteria-toxic elements [28,29], cryptic transcription [30], splicing [31], recombination [32], and unexpected mutations such as insertions or deletions [33]. As a result, viruses often fail to be rescued even when full-length cDNA clones are successfully assembled. In recent decades, advancements in modern molecular tools have helped overcome these challenges, making it easier to assemble infectious clones [34,35].

Infectious clones are divided into two types, RNA-launched and DNA-launched. RNA-launched types use bacterial expression promoters, such as the bacteriophage promoter SP6 and T7, located upstream of the viral genome, which allow the RNA to be transcribed in vitro [23]. The transcribed viral RNA genomes are then transfected into target cells, where they undergo translation, initiating virus replication and the synthesis of virions. These systems are highly efficient for rapidly producing large viral stocks at a high titer compared to DNA-launched systems. However, additional steps are required to transcribe from cDNA templates; sometimes, in vitro transcription could produce unexpected mutations in the transcribed RNA [36].

The second type, DNA-launched type infectious clones, is used in this study. DNA-launched systems use eukaryotic expression promoters, such as the CMV and simian virus 40 (SV40) promoters located upstream of the viral genome, enabling RNA transcription in live cells following DNA transfection, unlike RNA-launched systems that require in vitro transcription [17]. However, the CMV promoter has cryptic *E. coli* promoter (ECP) activity in bacteria, which induces genomic instability [36].

Bacterial artificial chromosome (BAC)-derived plasmids are widely used for generating infectious cDNA clones [37]. They are suitable for cloning large DNA fragments in synthetic low-copy number plasmids that stabilize large DNA insertions, thus preventing the expression of potentially toxic regions associated with their genome [38]. Infectious DNA clones based on BAC plasmids have been established for various RNA viruses, including coronavirus [39,40,41,42], influenza virus [43,44], and flavivirus [33,45]

In this investigation, a reverse genetic system was developed to generate synthetic viruses from genetically stable full-length infectious JEV3 cDNA. The CMV promoter was introduced upstream of the JEV3 genomic cDNA to transcribe genomic RNA and the HDVr sequence was added to the 3′ end to maintain the natural 3′terminus of JEV (Figure 1). Figure 2 demonstrates the successful retrieval and propagation of the recombinant virus within Vero cells, highlighting the sustained stability of the viral genome across successive passages. Additionally, a standard mutagenesis procedure was employed to introduce a specific point mutation into the BAC construct, serving as a genetic marker (Figure 3). These point mutations were introduced not only to distinguish the virus but also to facilitate the genetic engineering of the recombinant virus. The point mutation site was located at the C-terminus of the E gene. Our advantage of introducing point mutations in the E gene, as demonstrated in our JEV3 infectious clone model, is that the E gene can be easily replaced with any JEV E sequence of interest, including those from currently prevalent strains or even other flaviviruses [46]. Furthermore, the phenotypic characteristics of the recovered viruses were similar to those of the parental type, as confirmed by investigating the morphology of virus particles using cryo-EM. Both the parental and recombinant viruses exhibited similar morphologies (Figure 5). Mice were immunized with the same dose of inactivated parental and recombinant viruses. Moreover, we evaluated JEV-specific IgG titers and neutralizing antibodies. Both viruses produced antibodies and their neutralizing activities were also similar (Figure 6).

Currently, vaccination remains the primary approach for preventing JEV infection, given the absence of available antiviral therapeutics [47]. The current JEV vaccines rely on inactivated clinical isolates or attenuated clinical isolates serially passaged in cultured cells [48]. Recently, several genotype 3-based vaccines have been used to prevent JEV infection and have demonstrated adequate cross-reactivity against other genotypes [49,50]. However, vaccine approaches that depend on isolated strains struggle to adapt effectively to emerging variant strains. Furthermore, the rapidly evolving nature of RNA viruses has led to the emergence of novel variants, posing a challenge to the efficacy of existing vaccines [51]. Therefore, the introduction of innovative preventive vaccines that can address breakthrough infections caused by unexpected virus variants and provide coverage against diverse mutations is imperative [52].

In this study, we confirmed the generation of recombinant JEV3, which was stable in terms of its genome and exhibited similar growth kinetics, morphology, and immune responses in mice infected with the parent virus. The recombinant viruses can be easily genetically engineered and controlled, allowing the vaccine to adapt quickly to emerging variants. These advantages make our JEV3 recombinant virus more effective in protecting against new strains and various mutations compared to currently available vaccines. Overall, our findings significantly contribute to the ongoing advancements in JEV molecular virology by furnishing novel insights into JEV genome manipulation and vaccine development.

## Figures and Tables

**Table 1:** Information about the primers.

Primer	Sequence (5′–3′)
JEV3 Fragment 1 F	CTATATAAGCAGAGCTAGAAGTTTATCTGCGTGAACTTCTTG
JEV3 Fragment 1 R	CCAGTCAGTGATCAACTTTCCACTGTCAGTAGTGGTTCTGACC
JEV3 Fragment 2 F	CTACTGACAGTGGAAAGTTGATCACTGACTGGTGCTGTCGC
JEV3 Fragment 2 R	GGACAAATAATGAGCCAGCTTGTGAGTTAATTGAGGCTAGCG
JEV3 Fragment 3 F	AATTAACTCACAAGCTGGCTCATTATTTGTCCTGCCACGAGG
JEV3 Fragment 3 R	GCTGGTGGTGAGGAAGAACACAGGATCTGGGTCGGCATGGCATC
Silent F (*BstBl*)	CAAGTGTTTGGTGGTGCCTTTCGAACACTCTTCGGGGGAATG
Silent R (*BstBl*)	CATTCCCCCGAAGAGTGTTCGAAAGGCACCACCAAACACTTG
Detection (Envelope) F	TTCAACTGTCTGGGAATGGGCAATC
Detection (Envelope) R	AGCATGCACATTGGTCGCTAAGAAC

F and R indicate forward and revers primers, respectively.

## Data Availability

The data that support the findings of this study are available from the corresponding authors upon reasonable request.

## Supplementary Materials

The following supporting information can be downloaded at: https://www.mdpi.com/article/10.3390/vaccines12060597/s1, Figure S1. Agarose gel analysis of JEV3 amplicons. (A) Schematic representation of JEV3 cDNA fragments. Each fragment was amplified and then assembled into long fragments, F1, F2, and F3. (B-D) Agarose gel analysis of amplicon assemblies from Fragments 1, 2, and 3 (Figure 1): A+B+C (B), D+E (C), and F+G+H (D). M, marker; Neg, Negative; B, blank. Table S1. Information about the primers.
