# Supplementary material for: Insights into the Pathogenesis and Development of Recombinant Japanese Encephalitis Virus Genotype 3 as a Vaccine
Source: Vaccines (Basel). 2024 May 30;12(6):597. doi: 10.3390/vaccines12060597 (PMC11209496; doi:10.3390/vaccines12060597)
Supplement: Supplementary file 1 [file vaccines-12-00597-s001.zip › vaccines-2996120-supplementary.pdf]

Insights into the Pathogenesis and Development of Recombinant Japanese Encephalitis Virus Genotype 3 as a Vaccine

-Supplementary information-

Jae-Yeon Park[1], Hye-Mi Lee[1], Sung-Hoon Jun[3], Wataru Kamitani[4], Onnuri Kim[1], Hyun-Jin Shin[1,2*]

[1] College of Veterinary Medicine, Chungnam National University, Daejeon, 34134, Republic of Korea

[2] Research Institute of Veterinary Medicine, Chungnam National University, Daejeon, 34134, Republic of Korea

[3] Electron Microscopy & Spectroscopy Team, Korea Basic Science Institute, Cheongju, Chungcheongbukdo 28119, Republic of Korea

[4] Department of Infectious Diseases and Host Defense, Gunma University Graduate School of Medicine, Gunma, Japan

*Correspondence: Hyun-Jin Shin.

Laboratory of Infectious Diseases, College of Veterinary Medicine, Chungnam National University, Daejeon 34134, Republic of Korea; email: shin0089@cnu.ac.kr

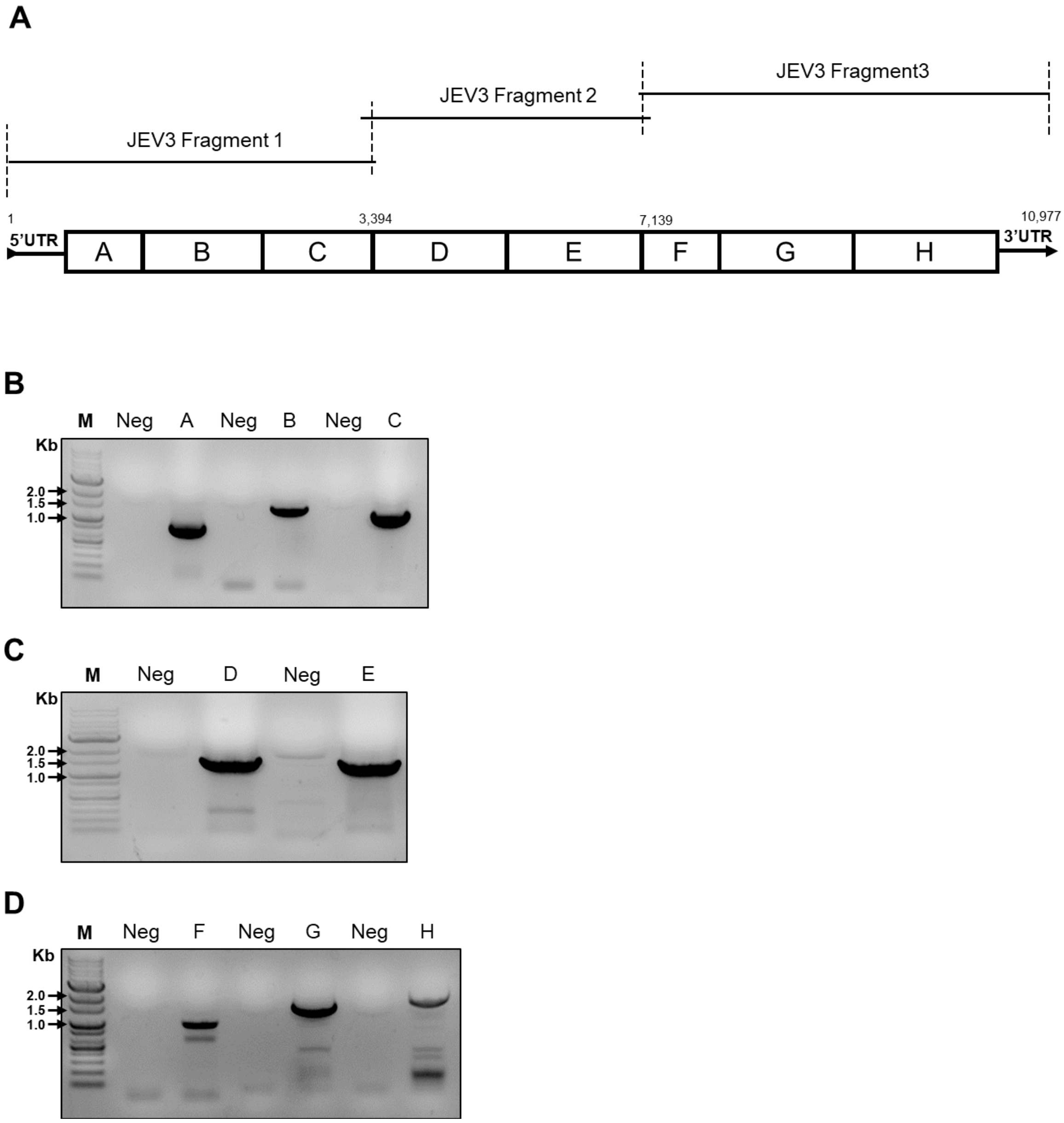

Figure S1. Agarose gel analysis of JEV3 amplicons. (A) Schematic representation of JEV3 cDNA fragments. Each fragment was amplified and then assembled into long fragments, F1, F2, and F3. **(B-D)** Agarose gel analysis of amplicon assemblies from Fragments 1, 2, and 3 **(Figure 1)**: A+B+C (B), D+E (C), and F+G+H (D). M, marker; Neg, Negative; B, blank.

Table S1. Information about the primers.

Primer	Sequence (5'-3')	Fragment location, nt
A F	AGAAGTTTATCTGCGTGAACTTCTTG	1-837
A R	GGTATCGTGTGGCTTTCGTTGAATCC	
B F	TCATGAAAACTGAGAACTGGATCG	838-2,258
B R	AAAGTCCCAGGCTGTGTCGCCCAACGC	
C F	GGCTCTATTGGAGGGGTTTTCAACTC	2,359-3,394
C R	TTTCCACTGTCAGTAGTGGTTCTGACC	
D F	GTTGATCACTGACTGGTGCTGTCGC	3,395-4,861
D R	AACCTCCATGGGCCTCCGTAAGCTATG	
E F	TGATCGAAAATGGAATGGAACAGATG	4,862-6,244
E R	CACACCGGAAGGTCAGCCGTCCTAAGC	
F F	GCTGGCCTACAAGGTGGCGTCCAATGG	6,245-7,139
F R	AGCTTGTGAGTTAATTGAGGCTAGCG	
G F	GGCTCATTATTTGTCCTGCCACGAGG	7,140-8,624
G R	AGTAGCCTTCACTTCATAGCTTCCGTG	
H F	GGCTCAGCCAGCTCTCTCGTCAATGG	8,625-10,977
H R	AGATCCTGTGTTCTTCCTCACCACCAGC	

F and R indicate forward and revers primers, respectively
